# LanceletDB: an integrated genome database for lancelet, comparing domain types and combination in orthologues among lancelet and other species

**DOI:** 10.1093/database/baz056

**Published:** 2019-05-18

**Authors:** Leiming You, Jiaqi Chi, Shengfeng Huang, Ting Yu, Guangrui Huang, Yuchao Feng, Xiaopu Sang, Xinhui Gao, Ting’an Li, Zirui Yue, Aijie Liu, Shangwu Chen, Anlong Xu

**Affiliations:** 1 School of Life Sciences, Beijing University of Chinese Medicine, Beijing, China; 2 State Key Laboratory of Bio-control, Guangdong Province Key Laboratory of Pharmaceutical Functional Genes, School of Life Sciences, Sun Yat-Sen University, Higher Education Mega Center, Guangzhou, China; 3 Department of Oncology, Dongfang Hospital, Beijing University of Chinese Medicine, Beijing, China

## Abstract

Lancelet (amphioxus) represents the most basally divergent extant chordate (cephalochordates) that diverged from the other two chordate lineages (urochordates and vertebrates) more than half a billion years ago. As it occupies a key position in evolution, it is considered as one of the best proxies for understanding the chordate ancestral state. Thus, the construction of a database with multiple lancelet genomes and gene annotation data, including protein domains, is urgently needed to investigate the loss and gain of domains in orthologues among species, especially ancient domain types (non-vertebrate-specific domains) and novel domain combination, which is helpful for providing new insight into the chordate ancestral state and vertebrate evolution. Here, we present an integrated genome database for lancelet, LanceletDB, which provides reference haploid genome sequence and annotation data for lancelet (*Branchiostoma belcheri*), including gene models and annotation, protein domain types, gene expression pattern in embryogenesis, different expression sequence tag sets and alternative polyadenylation (APA) sites profiled by the sequencing APA sites method. Especially, LanceletDB allows comparison of domain types and combination in orthologues among type species so as to decode the ancient domain types and novel domain combination during evolution. We also integrated the released diploid lancelet genome annotation data (*Branchiostoma floridae*) to expand LanceletDB and extend its usefulness. These data are available through the search and analysis page, basic local alignment search tool page and genome browser to provide an integrated display.

## Introduction

Chordates comprise three groups: the urochordates (sea squirts), cephalochordates (lancelets) and vertebrates (including the jawless lamprey and hagfish) (1–3). Lancelet (amphioxus), in particular, represents the most basally divergent living cephalochordate that diverged from urochordates and vertebrates 550 million years ago and retains a body plan and morphology most similar to fossil Cambrian chordates (1, 4). Analyses of the Florida lancelet genome (*Branchiostoma floridae*) have indicated that this chordate did not undergo two rounds of whole-genome duplications (2R-WGD) but rather shares extensive genomic conservation with vertebrates (5). Thus, lancelet occupies an evolutionary key position and has been widely used in research on cephalochordate biology and chordate evolution (6–9), especially the origin and evolution of vertebrate adaptive immunity (10–12). Recently, an active RAG transposon containing *ProtoRAG* (a prototypic recombination-activating gene or RAG), was discovered in the lower chordate lancelet. This sequence encodes *RAG1*-like (L) and *RAG2*L proteins and meets the structural criteria for the long-sought RAG transposon, illuminating the origins of V(D)J recombination and providing strong evidence in favour of the RAG transposon hypothesis for the origins of jawed vertebrate adaptive immunity (13–15). Since lancelet has become one of the best proxies for understanding the evolution of chordates and the origin of vertebrates, the construction of a database with multiple lancelet genomes and annotation data, including domain types, is urgently needed to investigate the loss and gain of domains in orthologues among species. In particular, we especially wanted to enable searching the ancient domain types (non-vertebrate-specific domains) and novel domain combinations during evolution from invertebrates to vertebrates, which may provide new insights into the chordate ancestral state.

Based on the sequenced genome of the American Florida lancelet (*B. floridae*), the JGI Genome Portal lacks another lancelet species (*Branchiostoma belcheri*). In particular, it contains few next-generation sequencing (NGS) reads to support the transcript candidates and is lacking alternative polyadenylation (APA) sites discovered and identified using an NGS-based approach (16). Another cDNA resource, `*Branchiostoma floridae* Gene Collection Release 1’, mainly contains short cDNAs and 5′- and 3′-expressed sequence tags (ESTs) from the five developmental stages of Florida lancelet, and hence its impact is very limited (17). We previously produced extensive datasets for *B. belcheri*, a lancelet distributed widely along the Chinese coast (14, 18), to provide additional information on this important evolutionary niche.

Here, we construct and present a web-accessible genomic database, LanceletDB, for two popular lancelet species (*B. belcheri* and *B. floridae*). LanceletDB can provide integrated biological information for lancelets, along with search and analysis tools to explore these data. These allow comparison of domain types and combination in orthologues so as to decode the ancient domain types (non-vertebrate-specific domains) and domain combination in chordates. LanceletDB holds a haploid genome sequence for the Chinese lancelet *B. belcheri*, which was created from the original diploid assembly using the HaploMerger tool reported previously (18, 19). HaploMerger, an easy-to-use automated pipeline, can be used to reconstruct allelic relationships for polymorphic diploid genome assembly and quickly generate a reference haploid assembly. Thus, the haploid assembly adopted in LanceletDB may represent a better reference assembly for lancelet *B. belcheri*, because it maintains better sequence contiguity and continuity and benefits the subsequent gene predictions, structural variation detection and other annotation efforts. As a web-based database, LanceletDB provides convenient URL-based retrieval, browsing and presentation of several types of information online, including genome sequences, gene models, gene function and domains in orthologues among type species, gene expression pattern in lancelet embryogenesis, various expression sequence tag (EST) sets and the APA sites profiled by the previously described NGS-based-sequencing APA sites (SAPASs) (20, 21). Additionally, we integrate the released diploid lancelet genome annotation data (*B. floridae*) to expand our LanceletDB and extend its usefulness. These data are available through the search and analysis page, basic local alignment search tool (BLAST) page and genome browser to provide an integrated display of annotation data.

## Materials and methods

### Generation of haploid genome sequence for *B. belcheri*

As described in our previous report (14, 18), the draft genome of the Chinese amphioxus *B. belcheri* was sequenced from an individual male, and using the Newbler and the Celera assembler (22–24), a polymorphic diploid assembly (with 4% heterozygosity) was generated from ~100× raw shotgun and paired-end reads that included both 454 FLX titanium reads (~30×) and Illumina 115-bp mate-pair reads (only for gap filling) (~70×). The HaploMerger package (18), an automated pipeline, was adopted to untangle allelic relationships in the generated soft-masked diploid assembly and further guide the subsequent creation of reference haploid assembly using the default parameters.

**Table 1:** Datasets listed by species in LanceletDB website

**Species**	**Datasets**	**Counts**	**Description and notes**
*B. belcheri*	B.belcheri_HapV2(v7h2)_genome	5679	Reference haploid genome assembly (v7h2) for *Belcheri’s* lancelet
*B. belcheri*	B.belcheri_HapV2(v7h2)_cds	35 293	Non-redundant transcript set for *Belcheri’s* lancelet genome (v7h2)
*B. belcheri*	B.belcheri_HapV2(v7h2)_proteins	35 293	Non-redundant protein set for *Belcheri’s* lancelet genome (v7h2)
*B. belcheri*	B.belcheri_v18h27.r3_ref_genome	2307	Reference haploid genome assembly (v18h27) for *Belcheri’s* lancelet
*B. belcheri*	B.belcheri_v18h27.r3_ref_cds	37 646	Non-redundant transcript set for *Belcheri’s* lancelet genome (v18h27)
*B. belcheri*	B.belcheri_v18h27.r3_ref_protein	37 646	Non-redundant protein set for *Belcheri’s* lancelet genome (v18h27)
*B. belcheri*	B.belcheri_v7h2_polyA_*V.anguillarum*-infected-intestine^a^	51 931	Describing APA sites, 3'-UTRs and heterogeneous cleavage sites, in the intestine of lancelet infected by *V. anguillarum* or not
*B. belcheri*	B.belcheri_454EST_ *V.anguillarum*-infected-intestine	223 103	ESTs from intestine of *Belcheri’s* lancelet challenged with *V. anguillarum*, obtained by the 454-sequencer
*B. belcheri*	B.belcheri_454EST_intestine	170 667	ESTs from intestine of *Belcheri’s* lancelet, obtained by 454-sequencer
*B. belcheri*	B.belcheri_454EST_embryo-mix	1 097 418	ESTs in *Belcheri’s* lancelet embryogenesis, generated by 454-sequencer
*B. belcheri*	B.belcheri_454EST_gill	467 739	ESTs from gill of *Belcheri’s* lancelet, obtained by 454-sequencer
*B. belcheri*	B.belcheri_454EST_liver	451 959	ESTs from liver of *Belcheri’s* lancelet, obtained by 454-sequencer
*B. belcheri*	B.belcheri_454EST_xiamen-beihai-merged_adult	98 118	ESTs from *Belcheri’s* lancelet (Beihai and Xiamen, China), generated by 454-sequencer
*B. belcheri*	B.belcheri_sangerEST_xiamen_adult	4074	ESTs from *Belcheri’s* lancelet (Xiamen, China), obtained by Sanger sequencing
*B. tsing*	B.tsing_sangerEST_qingdao_adult	24 412	ESTs from *Tsing’s* lancelet (Qingdao, China), obtained by Sanger sequencing
*B. floridae*	B.floridae_ESTs_embryogenesis-gastrula	262 037	ESTs from gastrula in *Florida* lancelet embryogenesis, obtained by Sanger sequencing
*B. floridae*	B.floridae_v1.allmasked^b^	3032	Reference diploid assembly for *Florida* lancelet
*B. floridae*	B.floridae_v1_anno.transcripts^b^	50 815	Transcript model for *Florida* lancelet
*B. floridae*	B.floridae_v1_anno.proteinsa^b^	50 815	Protein model for *Florida* lancelet

^a^Generated APA dataset integrated into our APASdb (http://genome.bucm.edu.cn/utr)

^b^Released datasets from JGI site.

### Gene prediction and functional annotation

The prediction of protein-coding gene models, including the functional annotation of corresponding proteins, was described in our previous publication (14).

### APA site annotation

The SAPAS method reported previously (20, 21), capable of high-throughput sequencing 3'-ends of polyadenylated transcripts, was adopted to identify and annotate polyadenylation sites for lancelet. In short, the total RNAs extracted from the intestinal tissues of *Belcheri’s* lancelets, which were challenged with *Vibrio anguillarum* or not, were used to prepare SAPAS sequencing libraries. Everything was the exact same as what was described previously (21). After sequencing, utilizing the same computational pipeline developed previously, the obtained SAPAS raw reads were processed to accurately map and quantify the usage of various poly(A) sites on a genome scale. The generated polyadenylation site datasets were used to annotate the APA sites for lancelet, including the APA sites that support the predicted transcript candidates.

**Figure 1:**
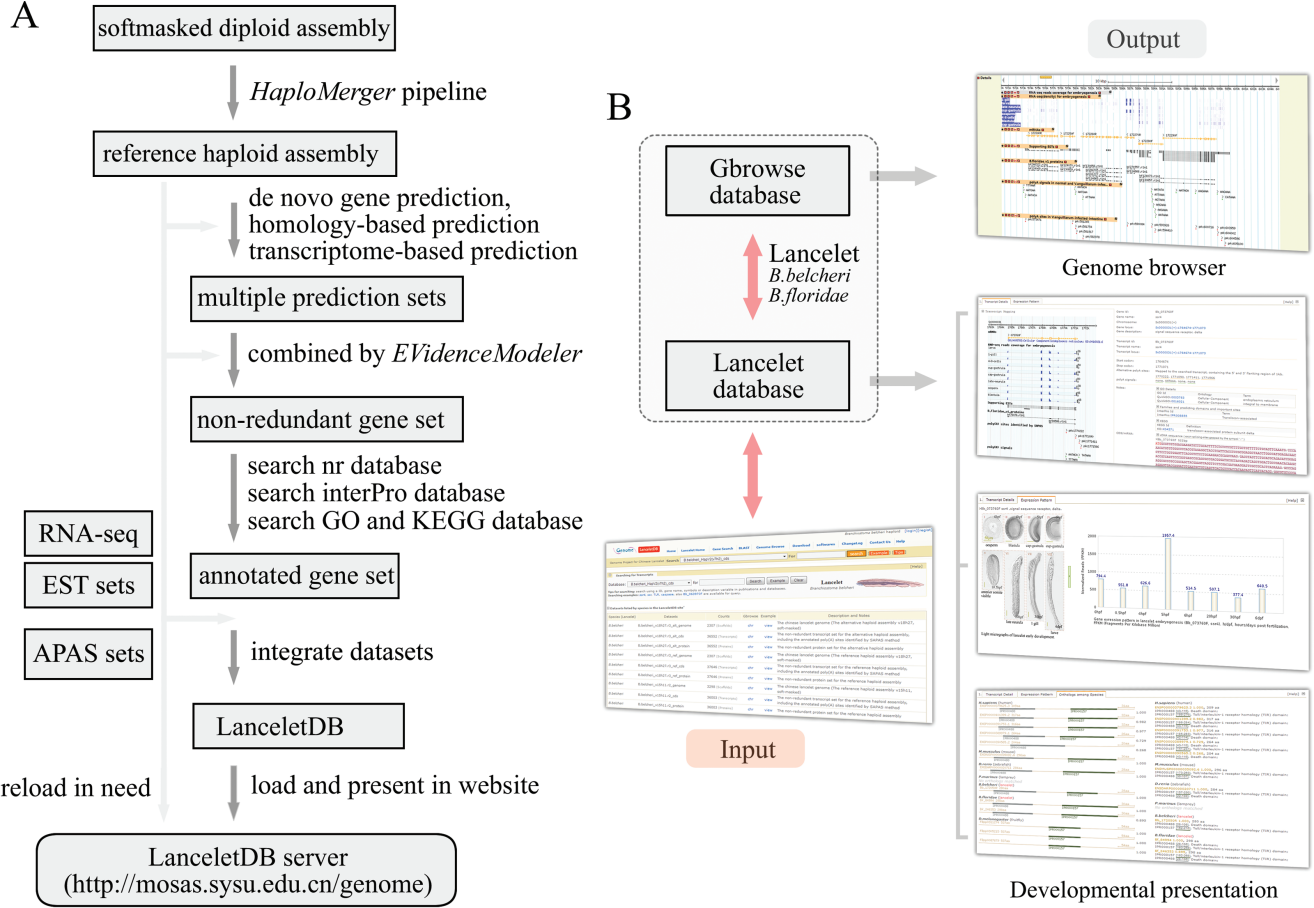
Overview of LanceletDB website. (A) Outline of LanceletDB building pipeline. Data flow is indicated by arrowed lines. (B) Architecture of LanceletDB website. Arrows denote direction of information flow, and several output pages are shown, including the popular genome browser (*Gbrowse*) and developmental presentations termed `*Transcript Detail’*, `*Expression Pattern*’ and `*Orthologues among Species’*.

### RNA sequencing

Multiple Chinese *Belcheri’s* lancelets in different developmental stages such as oosperm, 4–8 cells, blastula, cap gastrula, cup gastrula, late neurula, 1-gill slit and adult, including several adult tissues, were subjected to RNA sequencing (RNA-seq) using the Illumina GAIIx platform or the 454 FLX titanium platform. The obtained raw reads were filtered and mapped to the lancelet reference genome, termed as `*B.belcheri*_HapV2(v7h2)_genome’ (Table 1), using the Bowtie2 (version 2.2.9, default parameters) and TopHat packages (version 2.1.0, default parameters) (25, 26). The transcript analysis of mapped reads was performed to quantify the expression of gene models using the popular tool Cufflinks (version 2.2.1, parameters: -p 40 --time-series --multi-read-correct --library-type fr-unstranded --dispersion-method poisson) (25, 27).

### Database and website design

Based on the generated reference haploid assembly and related annotation information, including the annotated genes and proteins, APA sites and additional RNA-seq and ESTs data, the LanceletDB website was developed with open-source technologies (Figure 1A). The genome sequences, annotated transcript and protein datasets, APA site datasets and RNA-seq data were integrated to facilitate the query and display of genes in our website (Table 1). For example, the created searching dataset, named *B.belcheri*_HapV2 (v7h2)_cds, includes a total of 35 293 annotated gene models from Chinese *B. belcheri*. Various information regarding location, exon-intron structure and expression pattern of genes, poly(A) signal, poly(A) sites and 3′-untranslated regions (3′-UTRs), as well as the corresponding protein annotation including the domains, GO and KEGG, in the LanceletDB, are stored in a relational database using MySQL. The web-based HTML interactive interfaces combined with Java, Perl and PHP scripts can provide access to the database. GD modules of PHP, Bioperl modules and R modules are used for dynamic and graphical representation.

**Figure 2:**
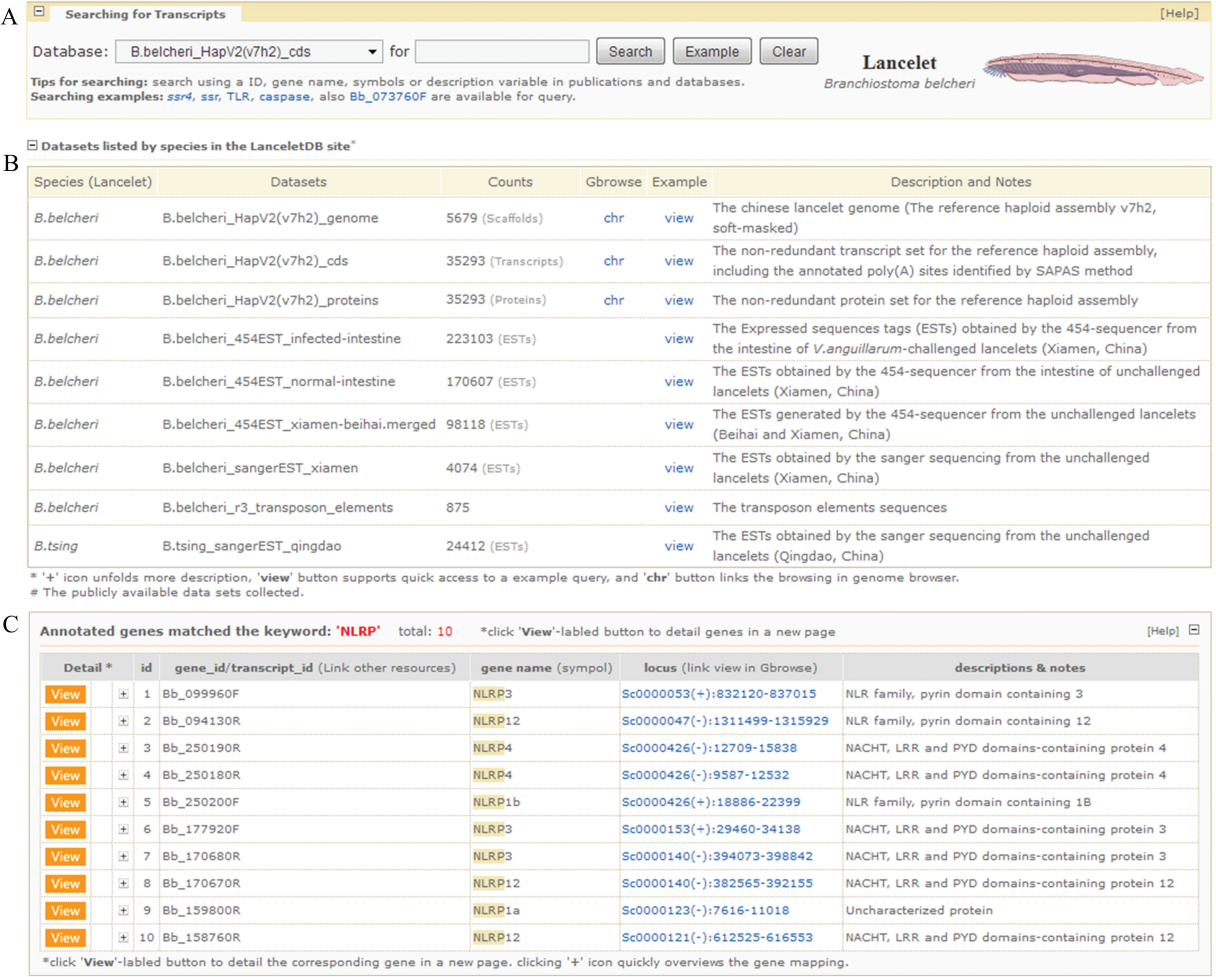
Screenshot of searching page and media page resulting from fuzzy query keyword of ‘NLRP’. (A) User retrieval interface designed to query datasets. (B) Descriptive list of datasets in retrieval interface. The list summarizes released datasets and directs user query. The ‘view’ button supports quick access to an example query of dataset and the ‘chr’ button links browsing of the dataset in a genome browser (Gbrowse). (**C**) List of gene models matching fuzzy keyword of ‘NLRP’. Texts in ‘locus’ column can guide users to specified URLs to browse gene models in genome browser. For the example mentioned here, it is available at http://genome.bucm.edu.cn/lancelet/search.php?seqkeywords=NLRP&db=Transcripts/B.belcheri_HapV2(v7h2)_cds.

## Results

### Datasets in LanceletDB

As listed (Table 1), currently, LanceletDB contains lancelet datasets involved in haploid genome sequences, annotated gene and protein models, APA sites, ESTs and RNA-seq data. These data are available and summarized in graphical representations on our LanceletDB website. Here, we use the searching dataset *B.belcheri*_HapV2 (v7h2)_cds for a detailed description. This dataset keeps a total of 35 293 non-redundant transcripts predicted for *Belcheri’s* lancelet. In addition, the corresponding annotation information for a gene model can be automatically matched, loaded and displayed in the interactive searching page, including the APA sites, gene expression pattern in embryogenesis, supporting ESTs and RNA-seq data and comparison of domain type and combinations between the lancelet gene model and its orthologues in other species such as fruit fly, lamprey, zebrafish, mouse and human. The released diploid genome (*B. floridae*) and annotation data were integrated to expand our LanceletDB. For the generated lancelet APA dataset named *B.belcheri*_v7h2_polyA_*V.anguillarum*-infects-intestine, it helps us to annotate the APA sites for the predicted transcript models in LanceletDB, but is also well documented as a new searching dataset in our APASdb (21), detailing APA site switching in the intestine of lancelets with and without challenge by *V. anguillarum.*

### General organization and access of LanceletDB

The general organization of the LanceletDB website is presented (Figure 1B), and the datasets are available from our LanceletDB web server at http://genome.bucm.edu.cn/lancelet. The complete set of predicted genes can be quickly queried and presented by a user’s keywords in the web-query interface, where the coding sequence (CDS) and protein sequence were loaded with annotation information such as gene name and description, GO, KEGG and domain types. The matched poly(A) sites and poly(A) signals are highlighted in the corresponding genome sequence to facilitate manual checks. The graphical user interface not only dynamically creates graphics to track APA sites and RNA-seq/EST coverage data together with the corresponding exon-intron structure of searched gene model, but also generates an additional two output pages to show the expression pattern of the corresponding gene model in lancelet embryogenesis, especially the comparison of domain types and combination between the searched lancelet gene model and its orthologues in other species. The generated datasets were integrated into a genome browser database, via the popular genome browser (Gbrowse) (28), to provide an interactive and graphical view of the genome, transcripts, APA sites and transcript annotations on a genome-wide scale.

### Searching LanceletDB

Our `*Gene Search’* feature is designed to search the complete dataset of predicted genes in LanceletDB and present gene information for a user’s genes of interest according to the searching tips. Currently, gene identifier (id) is allowed for precise query and fuzzy query using keywords such as gene name, symbol and simple description is permitted. Clicking the button labelled `Example’ can yield an example keyword suited for searching a selected dataset (Figure 2A), and the subsets and simple descriptions for the searched dataset can be found (Figure 2B). Fuzzy search, using the fuzzy keyword ‘NLRP’ (NLR family, pyrin domain containing), may lead to a media page to list all matched genes in a dynamic table (Figure 2C). This facilitates the selective view of their corresponding gene information in a linked detail page (described next), but searching with a precise keyword can give users quick access to the detailed page to view various types of information for the gene model and related graphics.

### Annotation and graphical display for a gene model

**Figure 3:**
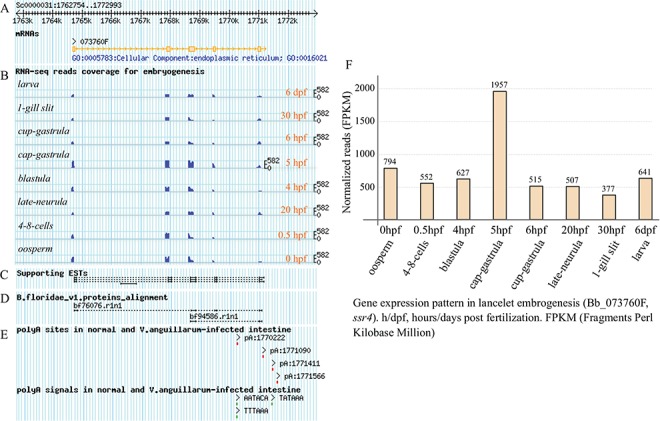
Exon structure of ssr4 gene model and its expression pattern during lancelet embryonic development. (A) Picture layer for tracking exon mapping. (B) Picture layer for tracking RNA-seq read mapping coverage. Reads generated from samples involved in lancelet embryogenesis, including oosperm (0 hpf), 4–8 cells (0.5 hpf), blastula (4 hpf), cap gastrula (5 hpf), cup gastrula (6 hpf), late neurula (20 hpf), 1-gill slit (30 hpf) and larve (6 dpf). (C) Picture layer for tracking ESTs to support gene model. (D) Picture layer for tracking BLAST alignment of Florida lancelet protein model to Belcheri’s genome. (E) Picture layer for tracking APA sites and poly(A) signals mapped to searched gene model. APA sites were identified by SAPAS method. (F) Bar chart indicating expression pattern of ssr4 during lancelet embryogenesis for 0 hpt to 6 dpf. Time points approximately correspond to major development stages such as oosperm, 4–8 cells, blastula, cap gastrula, cup gastrula, late neurula, 1-gill slit and larve. h/dpf, hours/days post fertilization; FPKM, Fragments per Kilobase Million. For direct browsing the example mentioned here, readers are asked to refer to http://genome.bucm.edu.cn/lancelet/search.php?seqkeywords=ssr4&db=Transcripts/B.belcheri_HapV2(v7h2)_cds.

Under the `Transcript Detail’ tab in the detailed page (Supplementary Figure S1), there is a summary for the queried gene model, including locus, gene name and description, as well as the APA sites and poly(A) signals mapped to the searched transcript locus (containing 5′ and 3′ flanking regions of 1 kb). The corresponding protein sequence is also loaded, and the exon splicing sites of the loaded protein CDS are gapped by the symbol `/’ to facilitate confirmation of the joint exons. The matched GO and KEGG annotations, including the domain types of protein model, are listed in several dynamic tables (Supplementary Figure S1, right). In particular, the APA sites, supporting ESTs and RNA-seq data and the exon-intron structure of the queried gene model are graphically presented at a proper scale, which enables tracking of them to the corresponding genome (Figure 3A–E). In addition, the exons, including the matched poly(A) sites and poly(A) signals, are highlighted in the corresponding genome sequence to facilitate a manual check (Supplementary Figure S2). Here, we take the lancelet gene model of signal sequence receptor 4 (*ssr4*) for example. The unfolded panel labelled `Transcript Mapping’ tracks the transcript structure of *ssr4*, together with the supporting ESTs mapping and RNA-seq read coverage during lancelet embryogenesis (such as oosperm, 4–8 cells, blastula, cap gastrula, cup gastrula, late neurula, 1-gill slit and larva). The BLAST alignment of the Florida lancelet *ssr4* protein to *Belcheri’s* lancelet genome is tracked to support the gene model. To track the APA-sites matched to the ssr4 model, four APA sites are detected (pA:1770222, pA:1771090, pA:1771411 and pA:1771566), including one poly(A) site (pA:1770222) located in the intron. The poly(A) sites (pA:1771090 and pA:1771566) appear to have no poly(A) signal, but each of the other poly(A) sites has at least a corresponding poly(A) signal (Figure 3A–E). To facilitate the manual checking of the cleavage sites in the corresponding genome sequence (unfolded panel labelled ‘Exons Structure’), all the detected heterogeneous cleavage sites clustered to a poly(A) site are underlined and highlighted in red, and their existing upstream poly(A) signals are highlighted in green. The searched transcript locus is indicated, including the marked exons (light grey background with a brown font). The most-frequently used cleavage site, defined as the reference poly(A) site in each cluster, is highlighted in dark red and underlined in bold (Supplementary Figure S2).

### Gene expression profile in lancelet embryogenesis

Clicking the `Expression Pattern’ tab on the detail page (Supplementary Figure S3) draws another matched graphic dynamically, i.e. a bar chart indicating the usage quantification of the queried gene in the major stages of lancelet embryogenesis. In addition, a group of micrographs (Supplementary Figure S3, left) are given to facilitate confirmation of the morphous of lancelet early development (oosperm, 4–8 cells, blastula, cap gastrula, cup gastrula, late neurula, 1-gill slit and adult). For the example query of *ssr4* (Figure 3F), the bar chart shows the usage quantification of the *ssr4* gene in the embryonic development of lancelet (from 0 hpf to 6 dpf). In general, the expression of the *ssr4* gene is high across all major stages of embryogenesis (FPKM value keeps more than 377). The expression of *ssr4* decreases first (0–0.5 hpf) but then increases (0.5–5 hpf) and reaches a maximum (FPKM value rises to 1957.4) at 5 hpf (cap gastrula stage), especially after a quick decrease at 6 hpf (cup gastrula stage), the *ssr4* expression appears to be stable (FPKM value keeps approximately 500).

### Domain types and combination of orthologues among species

**Figure 4:**
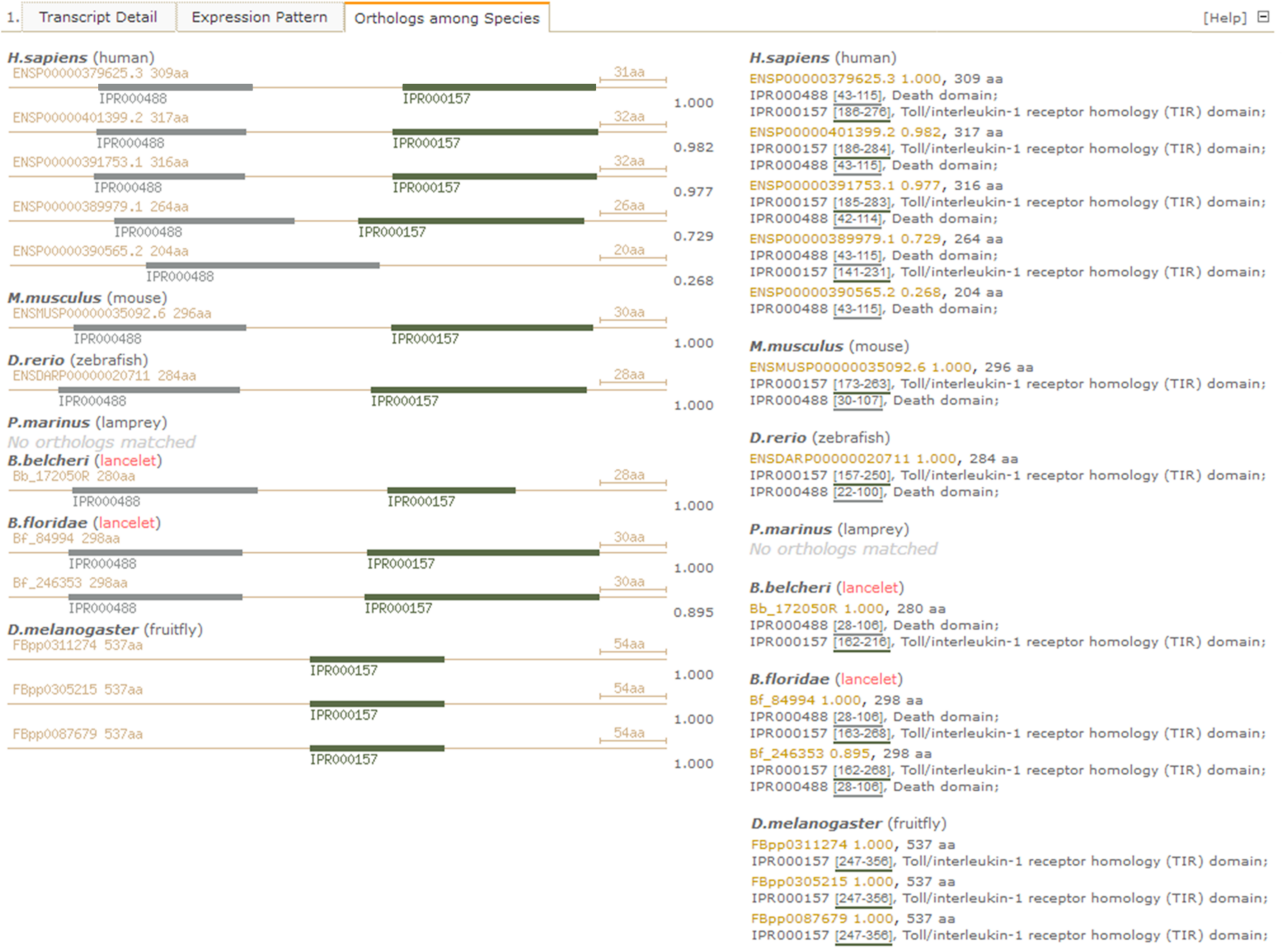
Screenshot of detail page with unfolded `Orthologues among Species’ tab to compare domain types and combination of myd88 orthologues between lancelet and other species. Pictures detail domain types and combination in identified myd88 orthologues among species (left). The myd88 orthologue ids (available in other resources) and length, including matched InterPro domains (ids, locus and description), are listed by species (right). IPR000488 labels death domain; IPR000157 labels Toll/interleukin-1 receptor homology (TIR) domain. For direct browsing the example described here, readers can refer to http://genome.bucm.edu.cn/lancelet/search.php?seqkeywords=Bb_172050R&db=Transcripts/B.belcheri_Hap V2(v7h2)_cds.

Under the `*Orthologues among Species’* tab in the detailed page (Figure 4), there is another summary for matched orthologues with high homology to the searched lancelet protein model in several types of species, such as fruit fly (*Drosophila melanogaster*), lancelet (*B. floridae*), lamprey (*Petromyzon marinus*), zebrafish (*Danio rerio*), mouse (*Mus musculus*) and human (*Homo sapiens*) (Figure 4, right). In addition, a set of pictures is generated to detail the predicted domains in these orthologues to provide direct comparison of domain types and locations among orthologues, enabling the investigation of loss and gain of domains, especially novel domain combination during evolution from invertebrate to vertebrate (Figure 4, left). Here, we take the lancelet protein model of *myd88* (myeloid differentiation primary response gene 88), for an example description (accession id ‘Bb_172050R’ in our LanceletDB). As shown, a set of pictures is created and labelled with the original protein id (available in other public resources). They not only show the length of different *myd88* orthologues among species but also present the domain types and location of each *myd88* orthologue. It’s clear that the *myd88* protein mainly consists of two types of domains in human, mouse, zebrafish and lancelet, including the death domain (InterPro: IPR000488) and another Toll/interleukin-1 receptor homology (TIR) domain (InterPro: IPR000157). Notably, the *myd88* orthologue containing only a death domain is matched in humans (Ensembl: ENSP00000390565.2), but all *myd88* orthologues matched in fruit fly (*D. melanogaster*) had only a TIR domain, which suggests that the death domain seems to be absent in the *myd88* orthologues of fruit fly. There is no *myd88* orthologue match in lamprey (*P. marinus*), possibly because the existing lamprey protein set (from the public *Ensembl* database) is limited.

### Dynamic and graphical browsing of gene models

Based on the integration of LanceletDB and a genome browser database, via the popular genome browser (Gbrowse) (28), the LanceletDB website provides dynamic browsing of gene models associated with genomes, APA sites and annotations on a genome scale (herein called the ‘*Genome Browse*’ feature). Under the ‘*Browser*’ tab (Supplementary Figure S4, top), the selective view in three layers, such as overview, region and details, is provided for tracking gene models, APA sites and supporting EST alignment and RNA-seq read coverage in the lancelet genomes, including *B. belcheri* and *B. floridae*. Gene model datasets and other datasets from these species can be quickly loaded and graphically browsed online by clicking the ‘Select Tracks’ button or ‘*Select Tracks*’ tab to select the corresponding checkboxes for datasets in the unfolded ‘Tracks’ panel (Supplementary Figure S4, bottom). It enables a dynamic overall picture of multiple datasets involved in gene models, EST mapping, RNA-seq coverage and APA sites. Here, we give an example description for the lancelet gene model of Mitogen-activated protein kinase organizer 1 (*MORG1*) with the accession id 088770F (or Bb_088770F). As tracked and indicated in detailed layers, the exon structure of the *MORG1* model (mRNAs tracking panel), the BLAST mapping of ESTs supporting the *MORG1* model (ESTs tracking panel) and the RNA-seq read coverage for lancelet embryogenesis (RNA-seq tracking panel) are shown. The BLAST alignment of Florida lancelet *MORG1* protein to the *Belcheri’s* lancelet genome is presented to support the *MORG1* model. In particular, the APA sites (pA:425596, pA:425813 and pA:426003) mapped to the *MORG1* locus (containing the 5' and 3' flanking regions of 1 kb) are displayed, but there appears to be no poly(A) signal <24 nt upstream of poly(A) sites (Supplementary Figure S4, middle).

### LanceletDB BLAST

BLAST is a powerful tool for comparison of biological sequence data. LanceletDB BLAST (http://genome.bucm.edu.cn/lancelet/blast.php) tightly integrates with the LanceletDB via the lancelet-specific datasets. Sequences or sequence ids can be used to identify lancelet gene models annotated with GO, KEGG, expression profile, domain types and APA site data. The lancelet datasets available for BLAST search are involved in the genome sequences, gene and protein model sequences, EST sequences and other sequences. The BLAST searching displays sequence alignment results with direct links to the LanceletDB gene page (denoted by E-icon) and genome browser page (denoted by M-icon), especially the APASdb page (denoted by U-icon) to detail the matched APA sites, enabling further investigation of APA site switching in the intestine of lancelets with or without challenge by *V. anguillarum*.

## Discussion

We present a comprehensive website database for a reference haploid genome and gene models for lancelet, the most basally divergent extant chordate, including *B. belcheri* and *B. floridae*. The EST mapping alignment and RNA-seq read mapping coverage data are used to support the actual gene models, especially RNA-seq reads generated from eight lancelet samples corresponding to the major stages of early embryonic development (oosperm, 4-8 cells, blastula, cap gastrula, cup gastrula, late neurula, 1-gill slit and adult). Moreover, based on the developed NGS-dependent 3′-end sequencing strategy, namely, SAPAS (20, 21), the generated APA datasets were used to annotate the APA sites for lancelet genes. Therefore, in a sense, we provide additional experimental support for the APA sites and gene models in LanceletDB.

Based on the reference haploid genome assembly generated by the HaploMerger pipeline (18), we predict gene models for *B. belcheri* lancelet and provide functional annotation for protein models, including GO, KEGG and domain annotation. In particular, Inparanoid (29), an algorithm and tool that finds orthologous genes, helps find the orthologues of lancelet gene models in other types of species such as human, mouse, zebrafish, lamprey and fruit fly. The protein domains in orthologues were identified using the package of InterProScan5 (30). Thus, LanceletDB can provide a direct comparison of domain types and combination in lancelet orthologues among species, which is helpful to investigate the loss and gain of domains in orthologues, especially novel domain combination during evolution from invertebrate to vertebrate.

Overall, LanceletDB holds multiple genome sequences and annotation data for the lancelet species, the best proxies for understanding the chordate ancestral state. As a user-friendly website database, LanceletDB will be an increasingly valuable resource for the genome research community, especially for decoding ancient domain types (non-vertebrate-specific domains) and domain combination in the chordates, providing new insights into the chordate ancestral state and vertebrate evolution.

### Data Access

All sequence data from the *Belcheri’s* lancelet genome project have been deposited in GenBank under accession code PRJNA214454. All EST and RNA-seq reads are deposited in the NCBI *Sequence Read Archive* (http://www.ncbi.nlm.nih.gov/sra) under accession numbers SRX137009, SRX137010, SRX137015, SRX344155 and SRX344156. The reference haploid assemblies for *B. belcheri* are available on our website.

## Supplementary Material

LanceletDB_Supplemental-Data_6b_R1_baz056

## Notes

**Database URL:** http://genome.bucm.edu.cn/lancelet
