# Supplementary material for: LanceletDB: an integrated genome database for lancelet, comparing domain types and combination in orthologues among lancelet and other species
Source: Database (Oxford). 2019 May 18;2019:baz056. doi: 10.1093/database/baz056 (PMC6526094; doi:10.1093/database/baz056)
Supplement: LanceletDB_Supplemental-Data_6b_R1_baz056 [file lanceletdb_supplemental-data_6b_r1_baz056.doc]

**Supplemental Information**

You L, *et al.*, LanceletDB: an integrated genome database for lancelet, comparing domain types and combination in orthologues among lancelet and other species.

**Supplementary Figures**


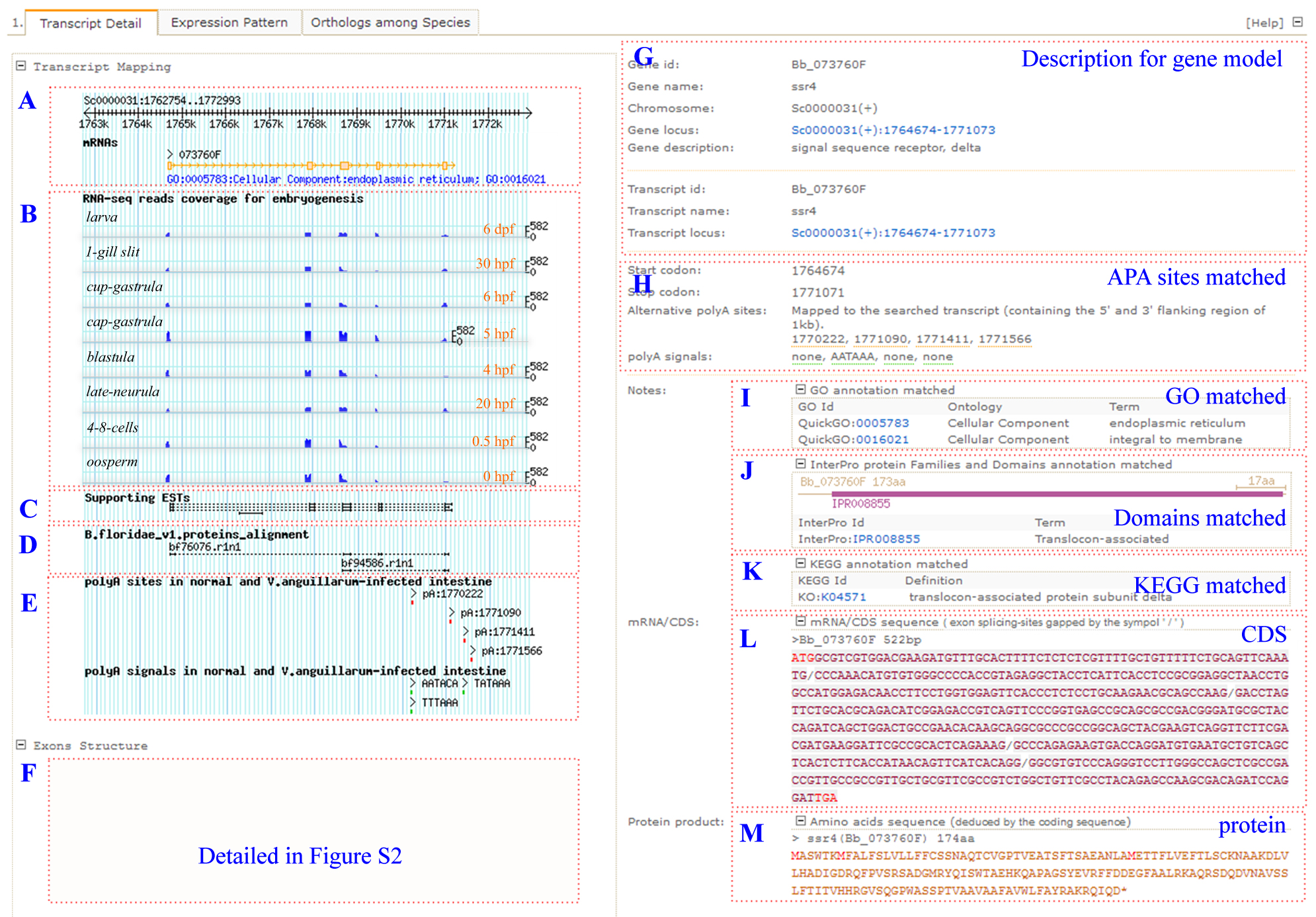


**Figure S1**. **Screen shot of the detail page with the unfolded ‘*Transcript Detail*’ tab to detail *ssr4* gene model**. (**A**) The created picture layer for tracking exons mapping. (**B**) The picture layer for tracking RNA-seq reads mapping coverage. Reads generated from samples involved in lancelet embryogenesis, including oosperm (0hpf), 4-8 cells (0.5hpf), blastula (4hpf), cap gastrula (5hpf), cup gastrula (6hpf), late neurula (20hpf), 1-gill slit (30hpf) and adult (6dpf). (**C**) The picture layer for tracking ESTs to support the gene model. (**D**) The picture layer for tracking blast alignment of Florida lancelet protein model to *Belcheri’s* genome. (**E**) The picture layer for tracking the APA sites and poly(A) signals mapped to the searched gene model. These APA sites were identified by the sequencing alternative polyadenylation sites (SAPAS) method. (**F**) Tracking exons structure of the *ssr4* gene model in genome sequence. Clicking the text ‘Exons Structure’ unfolds the tracking panel. This tracking will be detailed in **Figure S2**. (**G**) Description for the gene model of *ssr4*. (**H**) The matched APA sites mapped to *ssr4* gene model. (**I**) The matched GO annotation for *ssr4* gene model. (**J**) The matched domains in *ssr4* protein model. (**K**) The matched KEGG annotation for ssr4 model. (**L**) The protein coding sequence (CDS) for the ssr4 gene model. (**M**) The protein product corresponds to ssr4 gene model. For direct browsing the example here, it is avalilabe at [http://genome.bucm.edu.cn/lancelet/search.php?seqkeywords=*ssr4*&db=Transcripts/B.belcheri_HapV2(v7h2)_cds](http://genome.bucm.edu.cn/lancelet/search.php?seqkeywords=ssr4&db=Transcripts/B.belcheri_HapV2(v7h2)_cds).


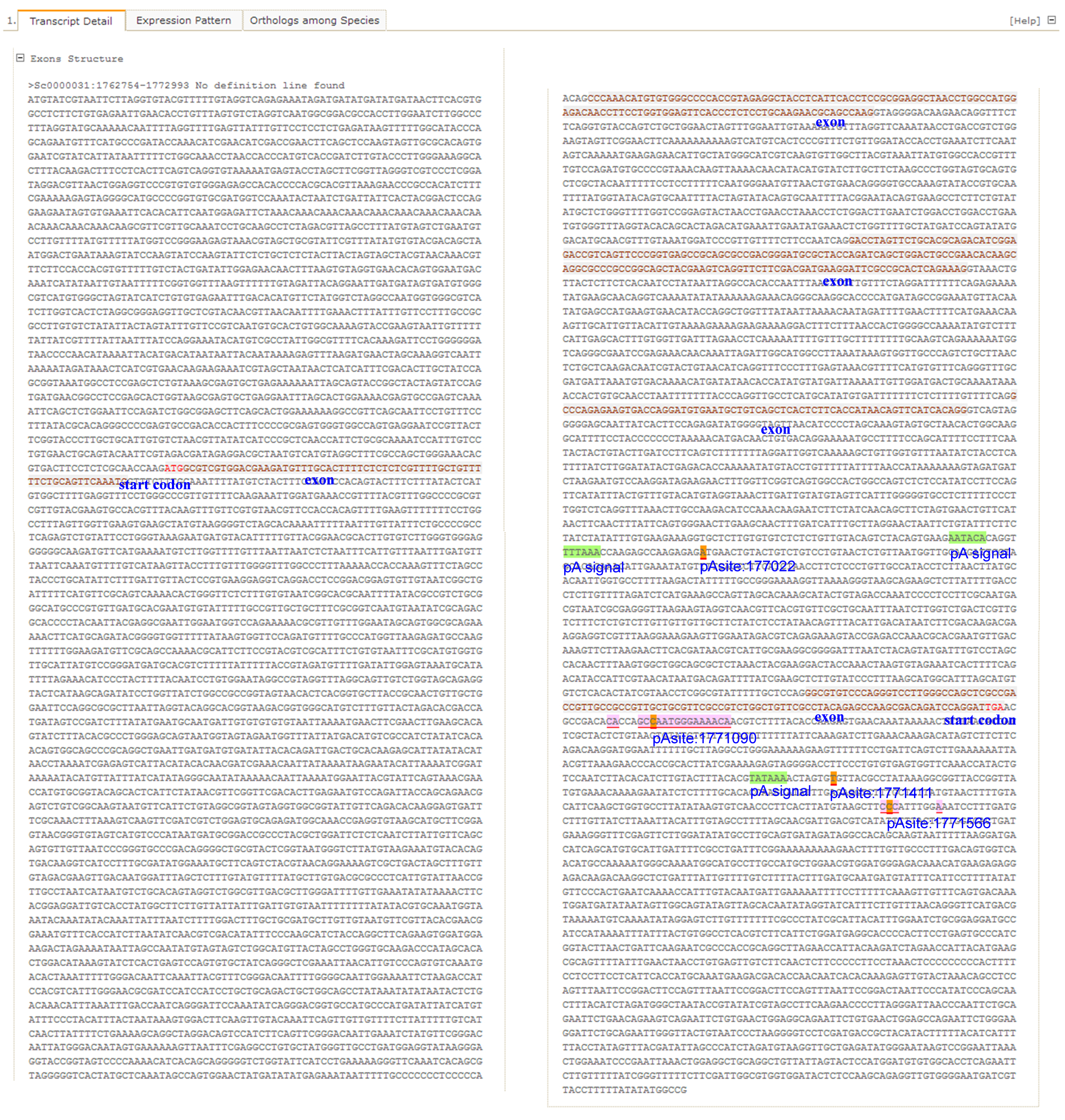


**Figure S2**. **Indication of exons structure of *ssr4* gene model in genome sequence**. Clicking the unfolded panel labeled ‘Exons Structure’ in the detail page, the exons, including the matched poly(A) sites and poly(A) signals, are highlighted in genome sequence. The searched transcript locus is indicated, including the marked exons (light gray background with a brown font). Four APA sites are detected (pA:1770222, pA:1771090, pA:1771411 and pA:1771566), including one poly(A) site (pA:1770222) located in the intron. The poly(A) sites (pA:1771090 and pA:1771566) seem to have no poly(A) signal, but each of the rest poly(A) sites has at least a corresponding poly(A) signal. The detected heterogeneous cleavage sites clustered to a poly(A) site are underlined and highlighted in red, and their upstream poly(A) signals if exists are highlighted in green. Especially, the most-frequently used cleavage site, defined as the reference poly(A) site in each cluster, is specially highlighted in dark red and underlined in bold. For direct view of the example mentioned here, readers are needed to refer to the address online at <http://genome.bucm.edu.cn/lancelet/search.php?seqkeywords=ssr4&db=Transcripts/B.belcheri_HapV2(v7h2)_cds>**.**


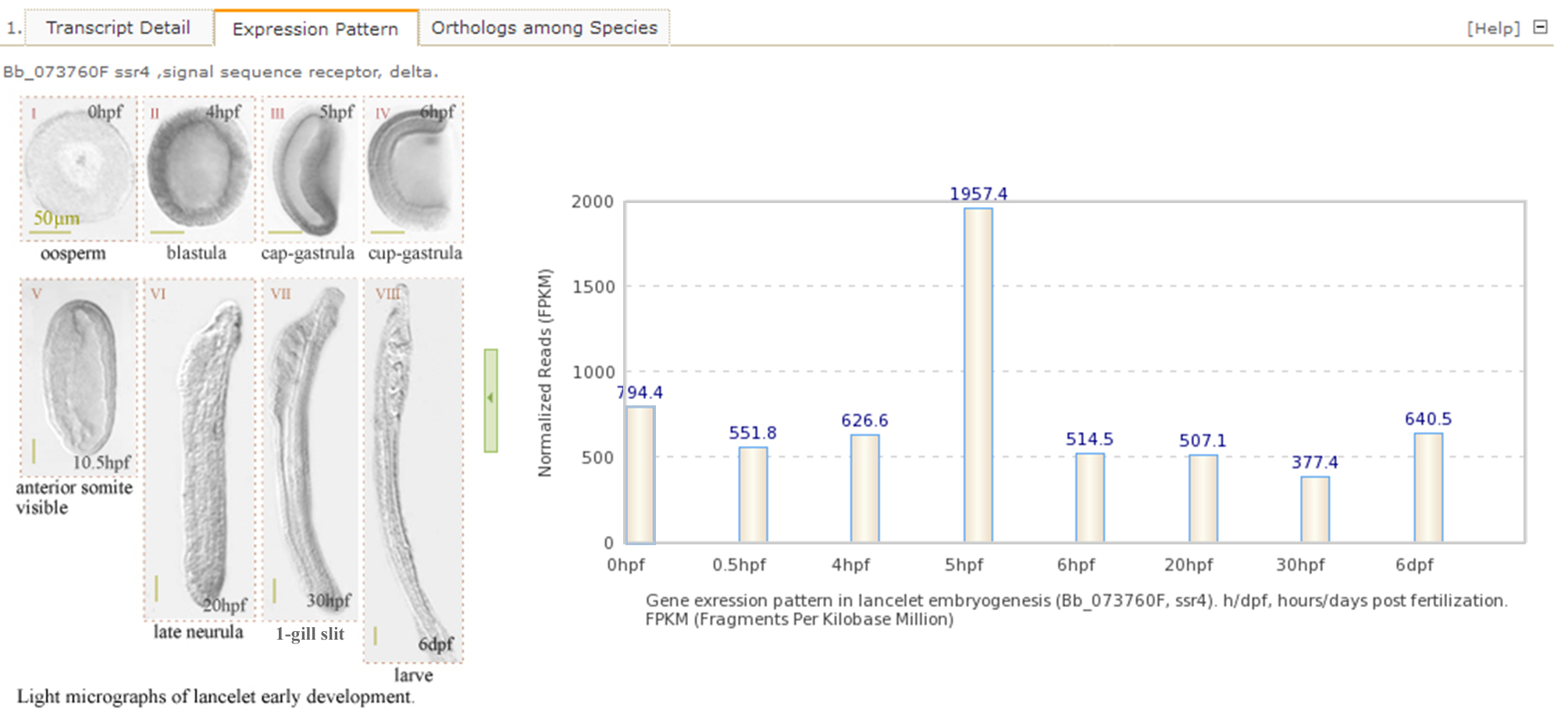


**Figure S3**. **Screen shot of the detail page with the unfolded ‘*Expression Pattern*’ tab to reveal the dynamic expression of *ssr4* in lancelet embryogenesis**. A set of micrographs, are given to facilitate checking the morphous of lancelet early development (oosperm, 4-8 cells, blastula, cap gastrula, cup gastrula, late neurula, 1-gill slit and adult) (**left**). The bar chart indicates the expression pattern of *ssr4* from 0 hpf to 6dpf (**right**). h/dpf, hours/days post fertilization. FPKM, Fragments Perl Kilobase Million. For the example mentioned, it is available online at <http://genome.bucm.edu.cn/lancelet/search.php?seqkeywords=ssr4&db=Transcripts/B.belcheri_HapV2(v7h2)_cds>**.**


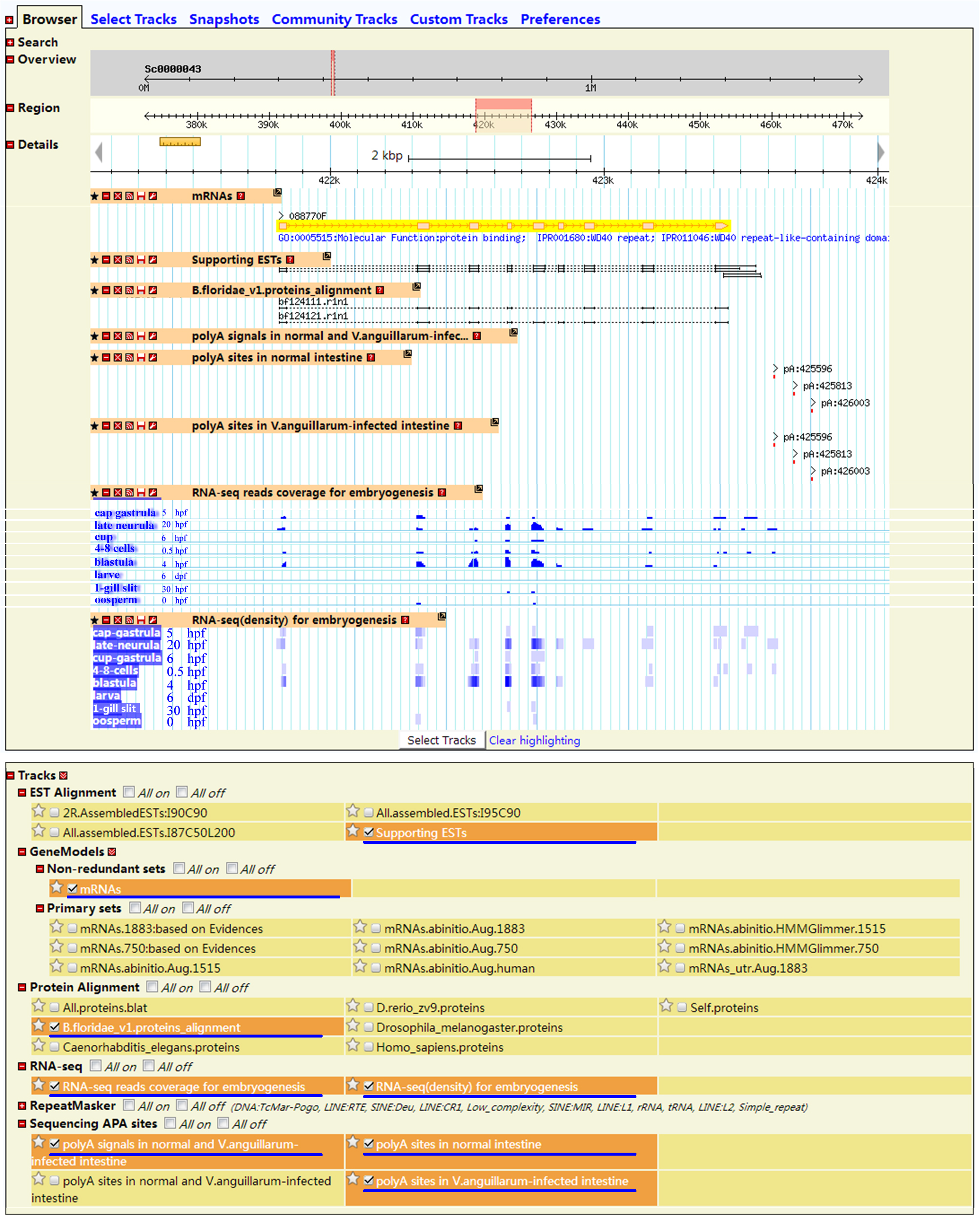


**Figure S4**. Screen shots of dynamic browsing of *MORG1* gene model associated with genome, APA sites and supporting EST/RNA-seq data by a genome browser (*Gbrowse*). *MORG1*, Mitogen-activated protein kinase organizer 1, is browsed by the genome browser (Gbrowse) with the lanceletDB accession id 088770F (or Bb_088770F). For direct browsing the *MORG1* gene model mentioned here online, the readers are asked to refer to <http://genome.bucm.edu.cn/lancelet/gbrowse.php?source=Branchiostoma.belcheri_HapV2(v7h2)&name=Sc0000043:418699-426567>.

**Supplementary Notes**

**Raw data access**

All sequence data from the *Belcheri’s* lancelet genome project have been deposited in GenBank under accession code *PRJNA214454*. All EST and RNA-seq reads are deposited in the NCBI *Sequence Read Archive* (SRA) (http://www.ncbi.nlm.nih.gov/sra) under accession number *SRX137009*, *SRX137010*, *SRX137015*, *SRX344155* and *SRX344156*. The reference haploid assemblies for *Branchiostoma belcheri* are available on our website (<http://genome.bucm.edu.cn/lancelet>).

**Gene prediction and functional annotation**

As previously reported , protein-coding gene models were obtained by integrating the results of *de novo* gene prediction, including homology-based and transcriptome-based prediction. Multiple prediction sets, including cDNA alignments by PASA , protein alignments by GeneWise , and RNA-seq alignments by Cufflinks , as well as the *ab initio* datasets from Augustus and GlimmerHMM and RNA-seq-based predictions by Augustus , were combined into a non-redundant gene set using EVidenceModeler . The initial combined prediction set was fed to Augustus for a new round of evidence-based prediction for alternatively spliced isoforms. Proteins were annotated by searching against the InterPro database , the Pfam domain database , the Gene Ontology (GO) database , and the KEGG database .

**References**

1. Huang, S., Chen, Z., Yan, X. *et al.* (2014) Decelerated genome evolution in modern vertebrates revealed by analysis of multiple lancelet genomes. *Nat Commun*, 5, 5896.

2. Haas, B.J., Delcher, A.L., Mount, S.M. *et al.* (2003) Improving the Arabidopsis genome annotation using maximal transcript alignment assemblies. *Nucleic Acids Res*, 31, 5654-5666.

3. Birney, E., Clamp, M. and Durbin, R. (2004) GeneWise and genomewise. *Genome Research*, 14, 988-995.

4. Trapnell, C., Williams, B.A., Pertea, G. *et al.* (2010) Transcript assembly and quantification by RNA-Seq reveals unannotated transcripts and isoform switching during cell differentiation. *Nature Biotechnology*, 28, 511-U174.

5. Stanke, M., Diekhans, M., Baertsch, R. *et al.* (2008) Using native and syntenically mapped cDNA alignments to improve de novo gene finding. *Bioinformatics*, 24, 637-644.

6. Majoros, W.H., Pertea, M. and Salzberg, S.L. (2004) TigrScan and GlimmerHMM: two open source ab initio eukaryotic gene-finders. *Bioinformatics*, 20, 2878-2879.

7. Haas, B.J., Salzberg, S.L., Zhu, W. *et al.* (2008) Automated eukaryotic gene structure annotation using EVidenceModeler and the program to assemble spliced alignments. *Genome Biology*, 9.

8. Hunter, S., Jones, P., Mitchell, A. *et al.* (2012) InterPro in 2011: new developments in the family and domain prediction database. *Nucleic Acids Res*, 40, D306-312.

9. Finn, R.D., Mistry, J., Tate, J. *et al.* (2010) The Pfam protein families database. *Nucleic Acids Research*, 38, D211-D222.

10. Ashburner, M., Ball, C.A., Blake, J.A. *et al.* (2000) Gene ontology: tool for the unification of biology. The Gene Ontology Consortium. *Nat Genet*, 25, 25-29.

11. Kanehisa, M. and Goto, S. (2000) KEGG: kyoto encyclopedia of genes and genomes. *Nucleic Acids Res*, 28, 27-30.
